# Impact of hospitalization duration before medical emergency team activation: A retrospective cohort study

**DOI:** 10.1371/journal.pone.0247066

**Published:** 2021-02-19

**Authors:** Jinmi Lee, Yujung Shin, Eunjoo Choi, Sunhui Choi, Jeongsuk Son, Youn Kyung Jung, Sang-Bum Hong

**Affiliations:** 1 Medical Emergency Team, Asan Medical Center, Seoul, Republic of Korea; 2 Department of Pulmonary and Critical Care Medicine, Asan Medical Center, University of Ulsan College of Medicine, Seoul, Republic of Korea; Kaohsuing Medical University Hospital, TAIWAN

## Abstract

**Background:**

The rapid response system has been implemented in many hospitals worldwide and, reportedly, the timing of medical emergency team (MET) attendance in relation to the duration of hospitalization is associated with the mortality of MET patients. We evaluated the relationship between duration of hospitalization before MET activation and patient mortality. We compared cases of MET activation for early, intermediate, and late deterioration to patient characteristics, activation characteristics, and patient outcomes. We also aimed to determine the relationship, after adjusting for confounders, between the duration of hospitalization before MET activation and patient mortality.

**Materials and methods:**

We retrospectively evaluated patients who triggered MET activation in general wards from March 2009 to February 2015 at the Asan Medical Center in Seoul. Patients were categorized as those with early deterioration (less than 2 days after admission), intermediate deterioration (2–7 days after admission), and late deterioration (more than 7 days after admission) and compared them to patient characteristics, activation characteristics, and patient outcomes.

**Results:**

Overall, 7114 patients were included. Of these, 1793 (25.2%) showed early deterioration, 2113 (29.7%) showed intermediate deterioration, and 3208 (45.1%) showed late deterioration. Etiologies of MET activation were similar among these groups. The clinical outcomes significantly differed among the groups (intensive care unit transfer: 34.1%, 35.6%, and 40.4%; *p* < 0.001 and mortality: 26.3%, 31.5%, and 41.2%; *p* < 0.001 for early, intermediate, and late deterioration, respectively). Compared with early deterioration and adjusted for confounders, the odds ratio of mortality for late deterioration was 1.68 (1.46–1.93).

**Conclusions:**

Nearly 50% of the acute clinically-deteriorating patients who activated the MET had been hospitalized for more than 7 days. Furthermore, they presented with higher rates of mortality and ICU transfer than patients admitted for less than 7 days before MET activation and had mortality as an independent risk factor.

## Introduction

Rapid response to general ward patients suffering from acute deterioration may not be possible owing to the presence of symptoms that are missed. However, regular monitoring and automated alert systems have been introduced to notify a medical emergency team (MET), which operates for 24 hours a day, to enact treatments and reduce instances of mortality [1, 2]. General ward patients experience unexpected adverse events in about 10% of cases, 7.3% of which are fatal [3]. The activation of the MET relies on changes in vital signs, and MET decides whether these patients need to be transferred to the intensive care unit (ICU) or the need for the discussion of do-not-resuscitate (DNR) orders with the patients’ family.

A few studies have reported that the timing of MET activation with respect to hospital admission is associated with patient mortality [4, 5]. Smith et al. showed that 27.3% of patients triggered MET after more than 7 days of hospitalization and that those patients had a higher rate of in-hospital mortality than patients who had been hospitalized for less than 7 days [4]. Further research is needed to comprehend the relationship between the timing of MET activation and length of hospital stay.

In this study, we investigate cases of MET activation for patients with early deterioration (less than 2 days after admission), intermediate deterioration (2–7 days after admission), and late deterioration (more than 7 days after admission) and associate them with patient characteristics, activation characteristics, and patient outcomes [6]. We also aim to determine the association, adjusted for confounders, between the duration of hospitalization stay before MET activation and patient mortality.

## Material and methods

### Study cohort

**Fig 1:**
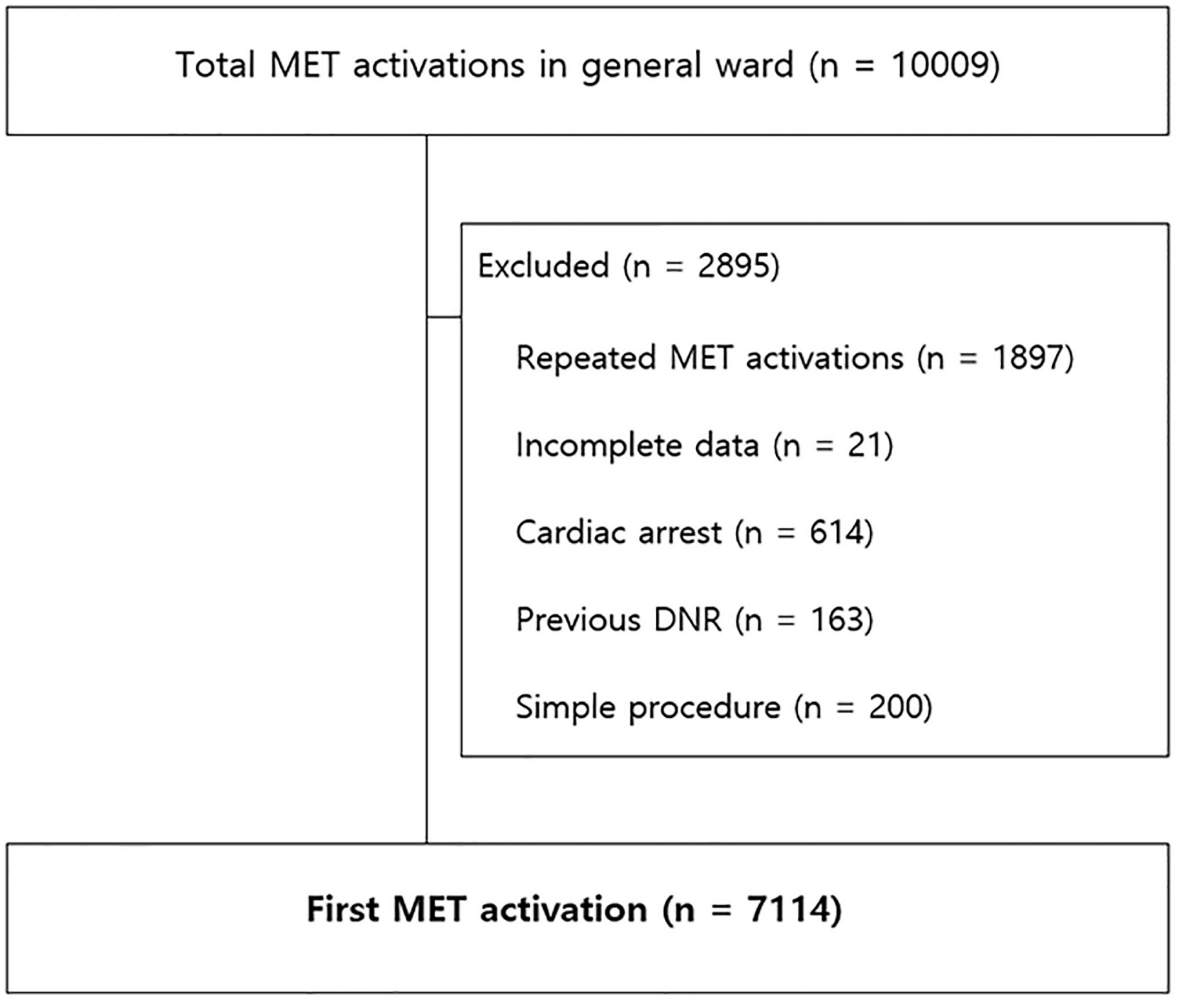
Flow chart of the inclusion of patients.

We retrospectively evaluated patients (>18 years) who triggered MET activation in general wards during the same hospitalization period (from March 2009 to February 2015) at a university-affiliated, tertiary care hospital with approximately 2700 beds (Asan Medical Center in Seoul, Korea), with the capacity for approximately 100,000 admissions (adult patients) per year. We excluded patients under 18 years, those with confirmed DNR orders, and cardiac arrest patients (Fig 1). According to the time when deteriorating patients activated the MET, we categorized as those with early deterioration (less than 2 days after admission), intermediate deterioration (2–7 days after admission), and late deterioration (more than 7 days after admission) [4].

### Description of the MET

Our hospital has a MET in place since 2008. The MET is available 24 hours every day, 7 days per week. It consists of three members of the ICU staff (intensivist), four ICU fellows, two internal medicine residents, and nine dedicated nurses with experience in critical care. At least one intensivist or fellow, one resident, and two dedicated nurses work on every duty [7]. The three types of MET triggers are as follows: 1) doctors, nurses, and other health care workers call the MET for help, or 2) the measurement of a patient`s vital signs and laboratory tests exceeded the pre-defined criteria in the electronic medical record-based automatic screening system, or 3) code blue was announced for cardiopulmonary arrest, as published previously [7, 8]. A MET is called by a general ward nurse or resident using dedicated MET numbers. The calling criteria for MET activation include crisis components based on the following physiological parameters: threatened airway, respiratory rate >30 breaths/min or <6 breaths/min, oxygen saturation <90% on the venturi mask 40% or O_2_ at a flow rate of 12 L/min, pulse rate <40 beats/min or >140 beats/min, systolic blood pressure <90 mm/Hg, sudden mental change [7]. The MET is a team that responds quickly and accurately to deteriorating adult hospitalized patients and its main activities are as follows: 1) interventions for vital signs stabilization in situations such as sepsis, shock, respiratory failure, and cardiac arrest, 2) advanced airway management (intubation for difficult airway, cricothyroidotomy), 3) device insertion for hemodynamic monitoring and checking point-of-care-testing (POCT) that includes arterial blood gas monitoring, chemistry, lactic acid and 4) determination of a treatment plan.

### Data collection

Patient characteristics, including age, sex, comorbidities, date and time of MET activation, clinical department at the time of MET activation, the type of trigger for MET activation, cause of MET activation, interventions during all activation, DNR orders before and after MET activation, and the result of MET activation (stay in general ward/transfer to ICU) are recorded after the MET activation. The causes of activation were categorized as follows: respiratory distress, sepsis, hypovolemic shock, anaphylactic shock, altered mental status, metabolic acidosis, or others (including hypotension, education for nurse, simple procedures). In addition, data on hospital mortality in the patients with MET activation were collected daily and inspected monthly [7]. The MET nursing staff reviewed electrical medical records (EMRs) for the patient characteristics, which were recorded during patient assessment and treatment using formal data extraction forms. The primary outcome was ICU transfer and hospital mortality. Furthermore, we included the first MET activation for each activation for analysis, as it was assumed that the first activation would shape the subsequent clinical course. Besides, previous studies also included first MET activation because recurrent clinical deterioration following MET activation in hospitalized patients is common and associated with an increased risk of ICU admission, length of hospital stay, and incidence of in-hospital mortality [9].

### Statistical analysis

The collected data were statistically analyzed using SPSS version 21 (IBM Corp., Armonk, NY, USA). We used univariate analyses to compare the intermediate deterioration group and the late deterioration group to the early deterioration group. Contingency tables were assessed using fisher’s exact test, and continuous variables that were not normally distributed were assessed using the wilcoxon rank sum test. Analysis of variance testing with bonferroni correction for multiple comparisons was used to determine the relationship between continuous and categorical variables. Multivariate logistic regression was used to determine the adjusted odds ratio of death in the hospital for patients in the intermediate and late deterioration groups by comparison with the early deterioration group [4]. We adjusted for age, sex, endotracheal intubation, and presence of underlying diseases. The results are presented as odds ratios (ORs) with 95% confidence intervals (CIs). *P*-values of less than 0.05 were considered statistically significant.

### Ethical considerations

The experimental plan used for this study received approval from the institutional review board of the Asan Medical Center (IRB No. 2016–1264). The study was conducted in accordance with the Korean Food and Drug Administration and the International Conference on the Harmonization of Good Clinical Practice guidelines. In the preparation of the care record form, the minimum amount of identifying information was used, and a new number was assigned to each patient for anonymity. The data collected through the care record forms were stored securely. The electronic data were stored in a computer with restricted access and encoded files. Only the registered research manager and co-researchers had access to the data.

## Results

### Characteristics of patients

A total of 7114 patients were included in our study. Of the 7114 patients who benefited from MET treatment, 1793 (25.2%) showed early deterioration (early group), 2113 (29.7%) showed intermediate deterioration (intermediate group), and 3208 (45.1%) showed late deterioration (late group). There was a significant difference in the distribution of patient departments (Table 1). Patients in the late group were significantly younger than those in the early group (median age: 64 years vs. 63 years; *p* < 0.001). The proportion of men in the late group was significantly higher than that in the early group (58.6% vs. 64.2%; *p* < 0.001). The proportion of patients with solid tumors in the intermediate group was significantly higher than that in the early group (43.2% vs.49.5%; *p* < 0.001), while the same proportion in the early group was significantly higher than that in the late group (43.2% vs. 40.2%; *p* = 0.040). The late group had a significantly higher proportion of patients with hematologic malignancy as the underlying disease than the early group (8.0% vs. 22.9%; *p* < 0.001) and a smaller proportion of chronic lung disease patients than the early group (13.6% vs. 10.1%; *p* < 0.001).

**Table 1:** Characteristics of patients.

Variable	Total (n = 7114)	Early deterioration group (n = 1793)	Intermediate deterioration group (n = 2113)	Late deterioration group (n = 3208)	*p*	*p*^a^	*p*^b^
Age, yr., median (IQR)	64 (53–72)	64 (53–73)	64 (53–73)	63 (52–71)	<.001	.931	.001
Sex, male, n (%)	4390 (61.7)	1050 (58.6)	1282 (61.7)	2058 (64.2)	<.001	.180	<.001
Illness category, n (%)					<.001^‡^	<.001^‡^	<.001^‡^
Medical, n (%)	5993 (84.2)	1613 (90.0)	1672 (79.1)	2708 (84.4)			
Oncology/Hematology	2224 (31.3)	451 (25.2)	590 (27.9)	1183 (36.9)			
Cardiology	105 (1.5)	20 (1.1)	34 (1.6)	51 (1.6)			
General medicine	3664 (51.4)	1142 (63.7)	1048 (49.6)	1474 (45.9)			
Surgical, n (%)	1121 (15.8)	180 (10.0)	441 (20.9)	500 (15.6)			
General surgery^†^	398 (5.6)	45 (2.5)	132 (6.2)	221 (6.9)			
Other surgery	723 (10.2)	135 (7.5)	309 (14.7)	279 (8.7)			
Underlying disease, n (%)							
Solid tumor	3108 (43.7)	774 (43.2)	1045 (49.5)	1289 (40.2)	<.001	<.001	.040
Hematologic- malignancy	1076 (15.1)	144 (8.0)	196 (9.3)	736 (22.9)	<.001	.169	<.001
Chronic lung disease	852 (12.0)	244 (13.6)	285 (13.5)	323 (10.1)	<.001	.913	<.001
Chronic heart disease	2710 (38.1)	689 (38.4)	780 (36.9)	1241 (38.7)	.405	.331	.858
Chronic liver disease	880 (12.4)	219 (12.2)	272 (12.9)	389 (12.1)	.702	.536	.927
Chronic renal disease	487 (6.8)	115 (6.4)	129 (6.1)	243 (7.6)	.136	.691	.127
DM	1656 (23.3)	425 (23.7)	452 (21.4)	779 (24.3)	.045	.084	.646

^†^ General surgery is a surgical specialty that focuses on abdominal contents including esophagus, stomach, small intestine, large intestine, liver, pancreas, gallbladder, appendix and bile ducts, and the thyroid gland,

^a^ Comparison between the early group and the intermediate group,

^b^ Comparison between the early group and the late group,

^‡^ Wilcoxon signed rank tests with Bonferroni correction (*p* < 0.025)

### Characteristics of MET activation

The characteristics of MET activation at the time of MET activation are shown in Table 2. MET activation occurred after a median of 6.0 days (interquartile range 2.0–16.0) after admission. MET activation was triggered automatically using only EMR-based screening criteria for 47.2% of patients, doctor’s call for 38.8% of patients, and nurse’s call for 14.0% of patients. Compared with the early group, the late group had a higher proportion of nurse-call-triggered activations (12.2% vs. 15.1%; *p* = 0.008). The most common cause of activation in all three groups was respiratory distress (51.4%). Sepsis was the reason for activation in 21.1% of cases in the early group compared with 14.7% in the intermediate group (*p* < 0.001). Hypovolemic shock was the cause of MET activation for 5.5% of cases in the early group compared with 8.2% in the intermediate group (*p* = 0.001). Anaphylactic shock was the reason for MET activation for 1.6% of cases in the early group compared with 0.8% in the intermediate group (*p* = 0.019) and 0.2% (*p* < 0.001) of cases in the late group. Altered mental status was the cause of MET activation for 5.1% of cases in the early group compared with 7.0% of cases in the intermediate group (*p* = 0.012) and 6.5% of cases in the late group (*p* = 0.040).

**Table 2:** Characteristics of MET activation.

Variable	Total (n = 7114)	Early deterioration group (n = 1793)	Intermediate deterioration group (n = 2113)	Late deterioration group (n = 3208)	*p*	*p*^a^	*p*^b^
Hospital days from admission to activation, median (IQR)	6 (2–16)	2 (1–2)	4 (3–6)	18 (11–30)	<.001	<.001	<.001
Activation type, n (%)							
EMR triggered	3362 (47.2)	838 (46.7)	1028 (48.7)	1495 (46.6)	.302	.233	.927
Doctor call triggered	2760 (38.8)	736 (41.0)	794 (37.6)	1230 (38.3)	.066	.027	.060
Nurse call triggered	993 (14.0)	219 (12.2)	291 (13.8)	483 (15.1)	.029	.066	.008
Activation cause, n (%)							
Respiratory distress	3656 (51.4)	915 (51.0)	1139 (53.9)	1602 (49.9)	.017	.073	.458
Sepsis	1379 (19.4)	379 (21.1)	311 (14.7)	689 (21.5)	<.001	<.001	.779
Hypovolemic shock	485 (6.8)	98 (5.5)	174 (8.2)	213 (6.6)	.002	.001	.099
Anaphylactic shock	53 (0.7)	29 (1.6)	17 (0.8)	7 (0.2)	<.001	.019	<.001
Altered mental status	448 (6.3)	91 (5.1)	148 (7.0)	209 (6.5)	.037	.012	.040
Metabolic acidosis	370 (5.2)	103 (5.7)	112 (5.3)	115 (4.8)	.367	.544	.162
Intervention, n (%)							
Endotracheal intubation	1210 (17.0)	268 (14.9)	352 (16.7)	590 (18.4)	.007	.145	.002
POCT^†^ (point of care testing)	4679 (65.8)	1129 (63.0)	1409 (66.7)	2141 (66.7)	.015	.015	.007
Vasopressor	731 (10.3)	180 (10.0)	193 (9.1)	358 (11.2)	.055	.337	.220
End of life care discussion	1089 (15.3)	223 (12.4)	326 (15.4)	540 (16.8)	.002	.151	<.001

^a^ Comparison of early group and intermediate group,

^b^ Comparison of early group and late group,

^†^ POCT (point of care testing) is measuring arterial blood gas monitoring, chemistry, lactic acid

Interventions carried out by the MET differed between groups: endotracheal intubation was implemented more often in the late group than in the early group (14.9% vs. 18.4%; *p* = 0.002). End-of-life care discussion was administered to 12.4% of patients in the early group, a proportion that was less than that of the intermediate group (15.4%; *p* = 0.151) and the late group (16.8%; *p* < 0.001).

### Outcomes of MET activation

The outcomes of MET activation are shown in Table 3. Clinical outcomes were significantly different among the three groups (ICU transfer, early: 34.1%, intermediate: 35.6%, late: 40.4%; *p* < 0.001; hospital mortality, early: 26.3%, intermediate: 31.5%, late: 41.2%; *p* < 0.001).

**Table 3:** Outcomes of MET activation.

Variable	Total (n = 7114)	Early deterioration group (n = 1793)	Intermediate deterioration group (n = 2113)	Late deterioration group (n = 3208)	*p*	*p*^a^	*p*^b^
ICU transfer, n (%)	2662 (37.4)	612 (34.1)	753 (35.6)	1297 (40.4)	<.001	.326	<.001
Hospital mortality, n (%)	2461 (34.6)	472 (26.3)	666 (31.5)	1323 (41.2)	<.001	<.001	<.001
Illness category					.008	.131	.001
Medical	2263 (92.0)	449 (95.1)	619 (92.9)	1195 (90.3)	<.001	.238	.004
Oncology/Hematology	1094 (44.5)	193 (40.9)	286 (42.9)	615 (46.5)			
Cardiology	32 (1.3)	4 (0.8)	11 (1.7)	17 (1.3)			
General medicine	1137 (46.2)	252 (53.4)	322 (48.3)	563 (42.6)			
Surgical	198 (8.0)	23 (4.9)	47 (7.1)	128 (9.7)	<.001	.936	.413
General surgery	106 (4.3)	11 (2.3)	22 (3.3)	73 (5.5)			
Other surgery	92 (3.7)	12 (2.5)	25 (3.8)	55 (4.2)			

^a^ Comparison between the early group and the intermediate group,

^b^ Comparison between the early group and the late group

The multivariate logistic regression analysis for hospital mortality is shown in Table 4. Compared with the early deterioration group, the odds ratio of hospital mortality for the intermediate group was 1.18 (*p* = 0.030), and 1.68 for the late deterioration group (*p* < 0.001). MET intervention of endotracheal intubation and underlying conditions of solid tumor, hematologic disease, and chronic liver disease were associated with higher in-hospital mortality.

**Table 4:** Multivariate logistic regression model for hospital mortality.

Variables	OR (95% CI)	*P*
First MET activation		
Early group	1.00	-
Intermediate group	1.181 (1.017–1.372)	.030
Late group	1.679 (1.462–1.927)	<.001
Sex		
Male	1.00	-
Female	.888 (0.795–0.992)	.036
Discussion of end of life care	5.748 (4.945–6.682)	<.001
Endotracheal intubation	2.167 (1.893–2.482)	<.001
Solid tumor	1.592 (1.409–1.800)	<.001
Hematology disease	2.915 (2.492–3.409)	<.001
Chronic liver disease	1.821 (1.554–2.134)	<.001
Chronic lung disease	.630 (0.533–0.745)	<.001

OR = odds ratio

Adjusted for age, sex, endotracheal intubation, and underlying disease

## Discussion

In this study, we found that acute deterioration after more than 7 days hospitalization period was an independent risk factor for in-hospital mortality and ICU transfer for both medical and surgical patients. The activation of the MET relies on changes in vital signs, and MET decides these patients transferring to ICU or discussing DNR orders.

Deteriorations that occurred after more than 7 days hospital admission accounted for >45% of the MET cases. Previous studies have attempted to explore the timing of MET activation in relation to admission and reported that approximately 30% of MET activations were triggered by patients that experienced late deterioration [4, 5]. Besides, our study demonstrated a higher mortality rate for patients with late deterioration than that shown by other studies (41% vs. 30%–32%) [4, 5].

Several factors could explain this independent association between hospital mortality and late deterioration. First, acute deteriorating cancer (oncology/hematology) patients accounted for 31.3% of the study population in this study, which is higher than the proportion in another study (5.8%) [4]. Cancer patients that were receiving chemotherapy, and those that underwent bone marrow transplantation, require long stays in the hospital; so, cancer patients had longer hospital stays and higher severity than patients with other diseases.

Second, more patients in the late deterioration group had received antimicrobial therapy than that in the other groups, suggesting that hospital-acquired infection was a contributing factor in some of these cases. “Hospital-acquired infection” often refers to sepsis, which is a major cause of exacerbation and death in cancer patients. Alp et al. showed that the mortality rate of hematological cancer patients with sepsis and septic shock was 80.5%, and septic shock was a major risk factor for mortality [10].

Third, there were more patients with DNR orders in the late group than in the early group. The number of DNR orders performed by METs reported by other studies ranged from 8% to 35% [11–19]. In this study, MET provided advice on end-of-life care for 15% of patients. Patients in the late group had more discussion of end-of-life care than patients in the early group, and this had the highest impact on mortality in the late group.

Regarding underlying disease, chronic lung diseases showed better prognoses than other underlying diseases. There are the National Early Warning Score 2 (NEWS2) or chronic respiratory early warning score (CREWS) in patients with pulmonary diseases. NEWS2 is useful in detecting deteriorating patients, which reflects hypercapnic respiratory failure, where items associated with oxygen saturation and oxygen supply are added, and CREWS is useful for patients with acute exacerbation of chronic obstructive pulmonary disease [20, 21]. As another example of a scoring tool, Kim et al. reported that the gastrointestinal early warning score (EWS-GI) may predict ICU transfer among patients admitted to gastroenterology wards [22]. Therefore, we suggest employing advanced cancer disease-related scoring tools to predict high risks of death associated with DNR orders.

This study has several limitations. First, we included first MET activation for each MET activation for analysis, as it was assumed that the first activation would shape the subsequent clinical course. Besides, previous studies also included first MET activation and the timing of MET activation has indicated that repeated MET activations have similar characteristics with the first MET activation [9]. Second, our study was performed in a single center with many cancer and transplantation patients. Hospitals that have different MET systems, different staffing models, different models of care, or a different mix of patients might produce different results. However, the observed incidence in our study was similar to that reported in the previous literature, and the MET activation of patients with early and late deterioration are likely to be common to other health organizations.

## Conclusions

Approximately half of the patients (both medical and surgical) with acute deterioration occurring more than 7 days after admission showed higher rates of hospital mortality compared to patients who had been in the hospital for less than 7 days. Factors that increase the mortality rate include cancer, hospital-acquired infections, and DNR orders. Interestingly, patients with chronic lung diseases were found to have a good prognosis regardless of the length of their hospitalization.

This is particularly important in situations of collaboration among the MET, the patient, and the general ward staffs to ensure that the underlying problem is correctly identified and an understanding of treatment goals is shared.

## Supporting information

S1 Checklist STROBE statement—Checklist of items that should be included in reports of observational studies. (PDF)

## References

[1] Jones DA, DeVita MA, Bellomo R. Rapid-response teams. N Engl J Med. 2011;365(2): 139–146. 10.1056/NEJMra0910926 21751906

[2] Devita MA, Bellomo R, Hillman K, Kellum J, Rotondi A, Teres D, et al Findings of the first consensus conference on medical emergency teams. Crit Care Med. 2006;34(9): 2463–2478. 10.1097/01.CCM.0000235743.38172.6E 16878033

[3] Lee BY, Hong SB. Rapid response systems in Korea. Acute Crit Care. 2019;34(2): 108–116. 10.4266/acc.2019.00535 31723915 PMC6786673

[4] Smith RJ, Santamaria JD, Faraone EE, Holmes JA, Reid DA, Tobin AE. The duration of hospitalization before review by the rapid response team: a retrospective cohort study. J Crit Care. 2015;30(4): 692–697. 10.1016/j.jcrc.2015.04.004 25981444

[5] Medical Emergency Team End-of-Life Care investigators. The timing of rapid-response team activations: a multicentre international study. Crit Care Resusc. 2013;15(1): 15–20. 23432496

[6] Lyons PG, Klaus J, McEvoy CA, Westervelt P, Gage BF, Kollef MH. Factors associated with clinical deterioration among patients hospitalized on the wards at a tertiary cancer hospital. J Oncol Pract. 2019;15(8): e652–e665. 10.1200/JOP.18.00765 31306039 PMC6694031

[7] Ahn JH, Jung YK, Lee JR, Oh YN, Oh DK, Huh JW, et al Predictive powers of the Modified Early Warning Score and the National Early Warning Score in general ward patients who activated the medical emergency team. PLoS One. 2020;15(5): e0233078 10.1371/journal.pone.0233078 32407344 PMC7224474

[8] Huh JW, Lim CM, Koh Y, Lee J, Jung YK, Seo HS, et al Activation of a medical emergency team using an electronic medical recording-based screening system*. Crit Care Med. 2014;42(4): 801–808. 10.1097/CCM.0000000000000031 24335439

[9] Stelfox HT, Bagshaw SM, Gao S. Characteristics and outcomes for hospitalized patients with recurrent clinical deterioration and repeat medical emergency team activation*. Crit Care Med. 2014;42(7): 1601–1609. 10.1097/CCM.0000000000000315 24670936

[10] Alp E, Tok T, Kaynar L, Cevahir F, Akbudak IH, Gündoğan K, et al Outcomes for haematological cancer patients admitted to an intensive care unit in a university hospital. Aust Crit Care. 2018;31(6): 363–368. 10.1016/j.aucc.2017.10.005 29429570

[11] Jones D, Moran J, Winters B, Welch J. The rapid response system and end-of-life care. Curr Opin Crit Care. 2013;19(6): 616–623. 10.1097/MCC.0b013e3283636be2 23799463

[12] Calzavacca P, Licari E, Tee A, Mercer I, Haase M, Haase-Fielitz A, et al Features and outcome of patients receiving multiple medical emergency team reviews. Resuscitation. 2010;81(11): 1509–1515. 10.1016/j.resuscitation.2010.06.017 20673606

[13] Casamento AJ, Dunlop C, Jones DA, Duke G. Improving the documentation of medical emergency team reviews. Crit Care Resusc. 2008;10(1): 29 18304014

[14] Jones DA, McIntyre T, Baldwin I, Mercer I, Kattula A, Bellomo R. The medical emergency team and end-of-life care: a pilot study. Crit Care Resusc. 2007;9(2): 151–156. 17536983

[15] Boniatti MM, Azzolini N, da Fonseca DL, Ribeiro BS, de Oliveira VM, Castilho RK, et al Prognostic value of the calling criteria in patients receiving a medical emergency team review. Resuscitation. 2010;81(6): 667–670. 10.1016/j.resuscitation.2010.01.025 20227811

[16] Downar J, Barua R, Rodin D, Lejnieks B, Gudimella R, McCredie V, et al Changes in end of life care 5 years after the introduction of a rapid response team: a multicentre retrospective study. Resuscitation. 2013;84(10): 1339–1344. 10.1016/j.resuscitation.2013.03.003 23499898

[17] Jäderling G, Calzavacca P, Bell M, Martling CR, Jones D, Bellomo R, et al The deteriorating ward patient: a Swedish-Australian comparison. Intensive Care Med. 2011;37(6): 1000–1005. 10.1007/s00134-011-2156-x 21369815

[18] Jones DA, Bagshaw SM, Barrett J, Bellomo R, Bhatia G, Bucknall TK, et al The role of the medical emergency team in end-of-life care: a multicenter, prospective, observational study. Crit Care Med. 2012;40(1): 98–103. 10.1097/CCM.0b013e31822e9d50 21926596

[19] Micallef S, Skrifvars MB, Parr MJ. Level of agreement on resuscitation decisions among hospital specialists and barriers to documenting do not attempt resuscitation (DNAR) orders in ward patients. Resuscitation. 2011;82(7): 815–818. 10.1016/j.resuscitation.2011.02.048 21482012

[20] Hodgson LE, Dimitrov BD, Congleton J, Venn R, Forni LG, Roderick PJ. A validation of the National Early Warning Score to predict outcome in patients with COPD exacerbation. Thorax. 2017;72(1): 23–30. 10.1136/thoraxjnl-2016-208436 27553223

[21] Echevarria C, Steer J, Bourke SC. Comparison of early warning scores in patients with COPD exacerbation: DECAF and NEWS score. Thorax. 2019;74(10): 941–946. 10.1136/thoraxjnl-2019-213470 31387892 PMC6817986

[22] Kim WY, Lee J, Lee JR, Jung YK, Kim HJ, Huh JW, et al A risk scoring model based on vital signs and laboratory data predicting transfer to the intensive care unit of patients admitted to gastroenterology wards. J Crit Care. 2017;40: 213–217. 10.1016/j.jcrc.2017.04.024 28445859

